# The Impact of Preoperative Inflammatory Markers on the Prognosis of Patients Undergoing Surgical Resection of Pulmonary Oligometastases

**DOI:** 10.3390/jcm9103378

**Published:** 2020-10-21

**Authors:** Francesco Londero, William Grossi, Orlando Parise, Jacqueline Cinel, Gianmarco Parise, Gianluca Masullo, Cecilia Tetta, Linda Renata Micali, Emanuela Mauro, Angelo Morelli, Jos G. Maessen, Sandro Gelsomino

**Affiliations:** 1 Cardiothoracic Department, Azienda Sanitaria Universitaria Integrata, 33100 Udine, Italy; francesco.londero@asufc.sanita.fvg.it (F.L.); william.grossi@asufc.sanita.fvg.it (W.G.); jacqueline.cinel@gmail.com (J.C.); gianluca.masullo@asufc.sanita.fvg.it (G.M.); angelo.morelli@asufc.sanita.fvg.it (A.M.); 2 Cardiovascular Research Institute, Maastricht University, 6229 ER Maastricht, The Netherlands; o.parise@icloud.com (O.P.); g.parise@maastrichtuniversity.nl (G.P.); l.micali@maastrichtuniversity.nl (L.R.M.); emanuelamauroam@gmail.com (E.M.); j.g.maessen@mumc.nl (J.G.M.); 3 Radiology Department, Rizzoli Institute, 40136 Bologna, Italy; cecilia.tetta@ior.it

**Keywords:** pulmonary oligometastases, surgery, neutrophils, C-reactive protein

## Abstract

The aim of this study was to assess the prognostic value of preoperative neutrophil-to-lymphocyte ratio (NLR) and C-reactive protein (CRP) levels in patients undergoing resection of pulmonary oligometastases. A retrospective analysis on 141 patients undergoing a first pulmonary metastasectomy in a single center was carried out. Two distinct analysis were performed subdividing patients according to their NLR ratio and CRP level. The main outcomes were survival and time to recurrence. At completion of follow-up 74 patients were still alive (52.5%). Subdividing patients according to their NLR yielded a significant difference in five-year progression-free survival (PFS, NLR < 4:32% vs. NLR ≥ 4:18%, *p* = 0.01). When subdivided by their CRP levels, patients with preoperative CRP < 5 mg/L demonstrated higher values of five-year overall survival (OS, 57% vs. 34%, *p* = 0.006) and five-year PFS (35% vs. 22%, *p* = 0.04). At multivariate analysis, level of neutrophils (*p* = 0.009) and lung comorbidities (*p* = 0.021) were independent predictors of death, whereas preoperative CRP (*p* = 0.002), multiple metastases (*p* = 0.003) and presence of lung comorbidities (*p* = 0.001) were independent predictors of recurrence. NLR and CRP are important predictors of prognostic outcome in patients undergoing pulmonary metastasectomy.

## 1. Introduction

Cancer is one of the leading causes of death worldwide. Although in some circumstances, the tumor can be resected with a radical purpose, patients may develop tumor recurrence and progression, with distant metastases which eventually lead the patient to death. In a minority of cases, such patients present with a “oligometastatic state”, defined as the presence of five deposits or less in no more than two distant organs [1]. In patients with pulmonary oligometastases, surgical metastasectomy is widely recognized as a valid therapeutic option [2]. Although this practice is carried out with the aim of prolonging survival or even being curative, most patients still develop systemic progression until death [3]. Several clinical features have previously been identified as prognostic factors, serving as a tool for selecting patients with better prognostic characteristics [3]. In the last two decades, increasing attention has been paid to the role of the immune system and the inflammatory state of cancer patients on the evolution of disease [4]. Within the complex network of inflammatory cells and cytokines in the tumor stroma, lymphocytes seem to play an important role in tumor control [4], while neutrophils may promote tumor growth and dissemination [5]. Indeed, the neutrophil-to-lymphocyte ratio (NLR) is an expression of the immunological balance of the host and has been shown to have a high prognostic value in many different cancer conditions [6]. In the context of oligometastatic lung disease, patients with higher neutrophil levels were found to have a worse outcome after radical resection [7,8]. However, the level of circulating leucocytes is not the only parameter that demonstrates a prognostic value in cancer patients: C-reactive protein (CRP) is an acute phase protein produced by the liver in response to activation of macrophages and T-lymphocytes during local and systemic inflammation. It has been emphasized that raised levels of CRP inversely correlate with survival in cancer patients and increase the risk of developing cancer in the overall population [9]. However, there is paucity of evidence regarding the influence of these two parameters on pulmonary oligometastases.

The aim of the present study is to assess the role of NLR and CRP on the prognosis of patients with lung oligometastases from several primary tumors.

## 2. Materials and Methods

This paper was structured according to the Strengthening the Reporting of Observational Studies in Epidemiology (STROBE) statement [10]. Approval of the study was waived by the ethical committee due to the retrospective nature of its design, according to national laws regulating observational retrospective studies (Italian law nr. 11,960, released on 13/07/2004). However, patients gave their written informed consent for the treatment of their data for scientific purposes.

### 2.1. Patient Population

We performed a retrospective revision of all clinical records of patients who underwent lung metastasectomy with curative intent between January 2008 and December 2017 in a single center (S. Maria della Misericordia University Hospital, Udine, Italy). In accordance with the traditional definition of oligometastasis [1], patients were deemed as oligometastatic and offered a surgical option when they presented with a limited number of deposits, amenable of radical resection with curative intent. However, in our institution we generally define as oligometastatic patients with no more than five metastases identified at radiological examinations. Moreover, the following indications for surgery were given at our local multidisciplinary meeting involving medical oncologists, radiologists, radiation oncologists and thoracic surgeons. Pulmonary metastases had to be considered suitable for complete resection and patients were expected to recover with good quality of life. Predicted postoperative forced expiratory volume in the first second (ppo-FEV1) and diffusion lung capacity for carbon monoxide (ppo-DLCO) > 40% were considered to be essential preconditions for surgery. Hylo-mediastinal nodal involvement must have been ruled out by preoperative radiological examination and, in case of suspected nodal involvement, a biopsy through endobronchial ultrasound (EBUS) or video mediastinoscopy had to be performed.

Inclusion criteria for the study were: (1) complete resection of primary tumor, (2) no other localizations of disease or extra-thoracic deposits amenable to local aggressive treatments, (3) histological confirmation of the metastatic nature of resected nodules, (4) no macroscopic residual disease, (5) no history of previous pulmonary metastasectomy for the same disease, (6) availability of preoperative blood sample test results, (7) no history of recent acute infections or inflammatory conditions within one month before surgery, and (8) no other active cancer. Bilateral or multiple metastases—defined as metastases involving both lungs or number of lesions > 1 were not considered to be exclusion criteria. Synchronous metastases, defined as metastatic lung nodules identified at time of discover of the primary tumor were not excluded from the study, as far as the primary tumor was radically resected before lung metastasectomy and restaging after primary tumor excision did not show further tumor progression in terms of number of deposits. Both open and minimally invasive techniques (video-assisted thoracic surgery—VATS) were employed to remove lung deposits.

Preoperatively, we collected data on patient features (age, gender, American Society of Anesthesiology (ASA) class, comorbidities, blood sample test analysis), oncologic aspects (disease-free interval (DFI), adjuvant/neoadjuvant treatment, number and size of pulmonary metastases), surgical modalities (approach, kind of resection), and outcomes (pattern of recurrence and survival, postoperative complications).

### 2.2. Inflammation Markers

Biochemistry analyses were performed on samples of peripheral blood obtained within one week before pulmonary metastasectomy. Blood samples were analyzed at the same institutional laboratory.

We collected data on blood cell values from peripheral blood sample tests and calculated the NLR by dividing the absolute number of neutrophils by the absolute number of lymphocytes. Patients were initially divided into two groups according to a NLR cut-off of 4, as described in a previous large meta-analysis [6].

We also collected data on preoperative CRP and albumin, but this latter was available in only a minority of patients and therefore the modified Glasgow prognostic score (mGPS), previously described in other studies [11], could not be calculated. We thus investigated only the role of CRP as a prognostic indicator. To assess the prognostic value of CRP, we used a cut-off value of 5 mg/L, which is also used in the mGPS, to subdivide the population into two groups.

The primary outcomes we investigated were overall survival (OS) and progression-free survival (PFS). OS was defined as the time interval between metastasectomy and death or censored event and PFS was defined as the time interval between metastasectomy and tumor recurrence or censored event. DFI was defined as the time interval between treatment of the primary tumor and development of metastases.

### 2.3. Statistical Analysis

Normality of distribution was assessed using the Kolmogorov–Smirnov test. Continuous data were summarized as mean and standard deviation for normal distributions and as median and 25th to 75th percentiles for non-normal distributions. Categorical variables were reported as frequencies and percentages. Comparisons were carried out using Fisher’s exact test and the McNemar test when appropriate. The Kaplan–Meier method and log-rank test were used for survival analysis. The Cox regression model was used to estimate predictors of death. Variables with a *p* value < 0.05 at univariate analysis were tested at multivariable analysis. The proportional hazard assumption was verified using Schoenfeld residuals. Cumulative incidence curves were used to graphically depict tumor recurrence, and statistical significance was tested with the Gray test. A competing risk analysis was used to avoid overestimation of the incidence of recurrence. Considering the long interval of patients’ recruitment, the survival and recurrence analysis was adjusted by year of surgery. Receiver operating characteristic (ROC) curve analysis was used to determine the optimal threshold for predicting death and tumor recurrence. We validated the results using the bootstrap method (1000 iterations). R, release 3.2.3 (R Foundation for Statistical Computing, Vienna, Austria) software, and “survival”, and “cmprsk” packages were used. Significance for hypothesis testing was set at the 0.05 two-tailed level.

## 3. Results

Median follow-up time was 35 months (IQR 23–59) and no patient was lost at follow-up. During this time interval, 207 patients underwent pulmonary metastasectomy with radical intent. Fourteen patients were excluded since they had a repeated metastasectomy, 10 were excluded from the study since they had experienced a preoperative inflammatory status, and 42 did not have a complete blood cell count available shortly before the intervention. Finally, 141 patients were included and represented the study population. Preoperative characteristics of patients are reported in Table 1.

All patients had obtained Eastern Cooperative Oncology Group (ECOG) performance status (PS) 0–1. No differences were found in rates of recurrence (*p* = 0.12) and survival (*p* = 0.15) between the different types of histology. When subdivided according to their NLR level, the two groups were comparable regarding baseline conditions, primary tumor origin, previous treatments (neoadjuvant or adjuvant treatments following resection of primary tumor), DFI and metastasis characteristics (size, number, synchronous/metachronous occurrence), whereas we observed a higher percentage of male patients in the CRP > 5 group, when patients where compared according to this parameter. Lung comorbidities were mainly represented by chronic obstructive pulmonary disease (COPD, 16/17 patients, 94.1%) and in one case by interstitial lung disease (5.9%). Information regarding the operative procedure is detailed in Table 2. The only significant difference between the two groups was a higher percentage of procedures performed through a minimally invasive approach in the NLR < 4 group (59.5% vs. 30%, *p* = 0.016) and in the CRP < 5 group, and in this latter we also observed a lower percentage of anatomic resection. No difference was found in the incidence of postoperative complications and length of stay between the two groups both stratified by NLR and CRP (Table 3). The 30-day mortality was 0%.

### 3.1. NLR

**Figure 1:**
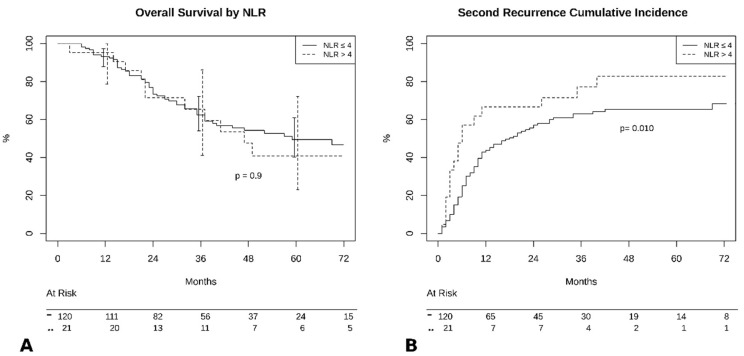
(**A**) Overall survival by neutrophil-to-lymphocyte (NLR) groups. (**B**) Cumulative incidence of recurrence by NLR groups.

At completion of follow-up, 67 patients (47.5%) died, and 74 were still alive (52.5%). Median survival time was 69 months (95% CI 45–93) in the NLR < 4 group and 47 months (95% CI 28–65) in the NLR ≥ 4 group (*p* > 0.05). Survival analysis showed no significant difference in 5-year OS (NLR < 4:50% vs. NLR ≥ 4:37%, *p* = 0.9, Figure 1A). However, there was a significant difference in 5-year PFS (NLR < 4:32% vs. NLR ≥ 4:18%, *p* = 0.01, Figure 1B). We also verified the reliability of the selected cut-off by calculating it in our population: using ROC curve analysis the optimal cut-off was 3.96 (AUC 0.73 (0.60–0.84). Since no patient had an NLR value falling in the interval 3.96–4.0 our results would not change by using the ROC curve derived cut-off.

### 3.2. CRP

**Figure 2:**
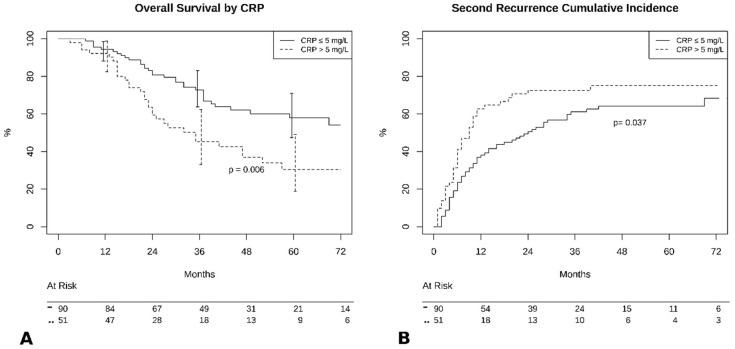
(**A**) Overall survival by C-reactive protein (CRP) groups. (**B**) Cumulative incidence of recurrence by CRP groups.

Patients with preoperative CRP < 5mg/L had better outcomes compared to those with higher values in terms of 5-year OS (57% vs. 34%, *p* = 0.006, Figure 2A), and 5-year PFS (35% vs. 22%, *p* = 0.04, Figure 2B).

### 3.3. Predictors of Outcome

Results of multivariate analysis are reported in Table 4**.** At multivariate analysis, level of preoperatory neutrophils (*p* = 0.018) and presence of lung comorbidities (*p* = 0.034) turned out to be independent predictors of death. Using ROC curve analysis, the optimal cut-off for level of neutrophils was 3640/mmc (AUC 0.697 (0.60–0.79)).

At competing risk regression analysis, levels of preoperative CRP (*p* = 0.003), multiple metastases (*p* = 0.004) and presence of lung comorbidities (*p* = 0.001) were independent predictors of recurrence. The optimal threshold of CRP at ROC curve analysis was 3.7 mg/L (AUC 0.674 (0.57–0.77)).

## 4. Discussion

In the last 20 years, increasing interest has been directed towards the complexity of the tumor microenvironment and the fundamental role of immune cells in regulating tumor cell growth and dissemination [4]. Apparently, neutrophils play a key role in this setting: the interaction between tumor cells and granulocytes stimulate the release of neutrophil extracellular traps (NET), a complex of web-like projections extending within the extracellular matrix (ECM) [12]. These protuberances are composed of nuclear DNA enriched in antimicrobial peptides that have shown good effectiveness in neutralizing pathogens both in vitro and in vivo [5]. However, although the pathogenic mechanism is not yet clearly understood, NET demonstrated a detrimental effect in a cancer microenvironment. This is likely due to the effect of peptides released from neutrophils in response to their contact with tumor cells: matrix metalloproteinases 9 (MMP-9), cathepsin-G, and neutrophils elastase (NE) have all been shown to promote degradation of the ECM, angiogenesis, vascular invasion and development of metastases [13,14,15].

A low NLR is associated with a more pronounced lymphocytic infiltrate at the margins of the tumor and with a better prognosis in patients with colorectal cancer [16]. This seems to confirm the correlation between level of circulating leucocytes and level of their presence at tumor sites, remarking the importance of assessing the level of blood cells as a proxy of their activity in the tumor microenvironment. Even though we did not assess the density of tumor-associated neutrophils (TAN), this parameter may indirectly be mirrored by the level of circulating cells [16], and the lower PFS observed in patients with a higher levels of circulating neutrophils found in the present study further supports the hypothesis of the pro-tumorigenic effect of granulocytes in the metastasis microenvironment. 

Several studies describe a detrimental effect of high NLR on survival in many cancer conditions: in a series of polymetastatic colorectal cancer (CRC) patients receiving palliative chemotherapy, NLR significantly correlated with OS, and the authors described how chemotherapy-induced normalization of this parameter resulted in survival improvement [17]. Cetin et al described similar results in a multicentric series of patients receiving chemotherapy for metastatic renal cell cancer [18]. In another study assessing the impact of NLR on tumor progression in patients undergoing curative resection of CRC, Ding et al found a negative effect of high NLR on recurrence-free survival (RFS) [19].

Even though our study found different rates of recurrence between patients with higher or lower values of NLR, we did not identify a significant difference in OS between the two groups. This finding might indicate a less pronounced effect of inflammation on the course of disease in the setting of oligometastatic cancers, as the prognostic role of NLR seems prominent in overt metastatic states [6]. Considering the well-known slow-progressing course of oligometastatic disease, another possible explanation of these results might be that the final effects of the observed increased rate of recurrence in patients with high NLR may be seen only with longer follow-up times. However, we observed that a value of preoperative neutrophils greater than 3640/mmc was a significant risk factor for death.

Conversely, our study demonstrated significantly different rates of recurrence and survival when patients were stratified according to their CRP levels. Furthermore, CRP turned out to be an independent risk factor for recurrence. Our results conform to those of Pastorino et al in documenting how the prognosis of patients undergoing resection of pulmonary metastases from various primary tumors is deeply influenced by values of CRP, measured preoperatively and three days after intervention [20].

CRP is the prototypical acute phase protein in humans, produced by the liver in response to interleukin 6 (IL-6) and interleukin 1β (IL-1β), which are mainly released in inflammatory sites by immune and endothelial cells [21]. It is an evolutionary conserved protein, since it has been found in all the studied species, from arthropods to humans [22]. In humans, CRP mediates several mechanisms of host defense from bacterial infection through complement activation, regulation of phagocytosis, and the binding of various antigens on the cell membranes [21]. CRP should, therefore, be considered not only a simple indicator of inflammation, but also a powerful weapon of the immune system. This is further confirmed by the observation that CRP shows a significant interaction with neutrophils: CRP is converted in its monomeric form (mCRP) in the inflammatory microenvironment, where it acts as a strong recruiter of granulocytes and inhibitor of apoptosis [23], eventually leading to further tissue damage and promotion of tumor growth. This mechanism not only confirms the role of both CRP and neutrophils in reducing the host’s tumor control, but also highlights the relevant effect of their interaction in promoting cancer progression.

In several previous studies, CRP has been used as a prognostic marker in a surrogated form, known as the modified Glasgow prognostic score (mGPS) [24]. This score, based on the values of preoperative CRP and albumin, was found to have a high prognostic value in many cancer conditions [24]. In our study, the mGPS could not be calculated due to the low percentage (31%) of patients with available preoperative albumin data. However, in the setting of oligometastatic disease, CRP seems to play a predominant role in predicting long-term survival and progressive disease [25].

Our investigation found that among patients undergoing surgical excision of pulmonary oligometastases the presence of lung comorbidities is an independent risk factor for earlier recurrence and death by any cause. In our study population, baseline lung diseases were mainly represented by COPD (94.1%). This result is coherent with the main findings of our study. COPD is a chronic inflammatory disease, which causes a rise in systemic inflammation markers, including CRP [26]. Nonetheless, one of the pathogenetic mechanisms leading to chronic lung inflammation is the imbalance between oxidative stress and antioxidant action [27]: granulocytes play a major role in this context, since in patients with COPD exacerbation, sputum neutrophils show a markedly increased production of reactive oxygen species (ROS) [28]. This, in our opinion, further confirms the strong relationship between local inflammation and cancer promotion even in the setting of pulmonary oligometastases and changes the perspective of inflammation from a simple cancer side effect to a major driver of tumor behavior.

Finally, our study highlighted how the presence of multiple metastases is a significant risk factor for further tumor recurrence. Our results are in accordance with those reported in two previous series of patients with lung metastases from CRC [29,30]. Several other studies described the presence of multiple metastases as a relevant risk factor for overall survival but its relevance in predicting tumor recurrence was not investigated [3,31,32]. Surgery, in the case of multiple lung nodules, is still an issue of large debate. The original definition of oligometastatic disease is based on a maximum number of five metastases in no more than two distant organs. However, the cut-off to distinguish an oligometastatic from a polymetastatic state has been chosen arbitrarily and based solely on the concept that, with few deposits, a radical resection could be achieved. If, on one hand, it is still considered reasonable to perform a surgical resection with radical intent in case of multiple metastases, on the other hand it should in our opinion be considered carefully in case other significant risk factors coexist. Evaluation of preoperative inflammatory markers may be strategic in this setting to discriminate patients who would benefit the most from an aggressive local treatment or wherein perioperative anti-inflammatory treatments could improve the systemic inflammatory pattern and potentially reduce the capability of tumor cells to further disseminate. To date, no study has investigated the application of these therapeutic regimens and their effectiveness in patients undergoing resection of lung metastases with radical intent. However, this will be the subject of our future research.

## 5. Conclusions

NLR and CRP are strong predictors of outcome in patients undergoing excision of pulmonary oligometastases from various primary tumors. Routine assessment of these parameters before posing a surgical indication may allow us to better stratify patients in terms of prognostic outcome and identify those who might benefit from perioperative systemic anti-inflammatory or targeted therapies, with potential considerable improvements in the overall outcome. More large-scale research is needed on this topic, with the potential of changing the paradigm of metastatic disease from an anteroom of inauspicious outcome to a curable disease through integration of local and systemic treatment.

## 6. Limitations

This study has several limitations: the results were obtained from a small retrospective series and several types of primary tumors have been included. Nonetheless, even if we ascertained the absence of acute pathologic conditions before surgery in our population, we could not rule out the coexistence of not-tumor-related subclinical inflammatory states that may have altered the preoperative markers values. Moreover, our short follow up time may have underestimated the real impact of NLR on survival. For the abovementioned reasons our results should be interpreted with caution.

## Figures and Tables

**Table 1:** Patient and tumor characteristics.

Population	All 141 (100)	NLR < 4 121 (85.8)	NLR ≥ 4 20 (14.2)	*p*	CRP < 5 90 (63.8)	CRP ≥ 5 51 (36.2)	*p*
**Gender:** Male	80 (56.7)	68 (56.2)	12 (60)	**0.81**	42 (46.7)	38 (74.5)	**0.002**
**Age at Surgery**	66 (IQR 59–71)	67 (IQR 59–70)	64.5 (IQR 59–73)	**>0.99**	67 (IQR 59–72)	64 (IQR 59–69)	**0.26**
**ASA:**							
2	94 (66.7)	82 (67.8)	12 (60)	**0.60**	61 (67.8)	33 (64.7)	**0.71**
3	47 (32.9)	39 (32.2)	8 (40)		29 (32.2)	18 (35.3)	
**Comorbidities**							
Coronaropathy	9 (6.3)	8 (6.6)	1 (5)	**>0.99**	6 (6.7)	3 (5.9)	**>0.99**
Arrhythmia	8 (5.7)	7 (5.8)	1 (5)	**>0.99**	3 (3.3)	5 (9.8)	**0.14**
Hypertension	52 (36.9)	43 (35.5)	9 (45)	**0.45**	29 (32.2)	23 (45.1)	**0.15**
Previous Cancer	34 (24.1)	28 (23.1)	6 (30)	**0.57**	17 (18.9)	17 (33.3)	**0.07**
Lung Disease	17 (12.1)	15 (12.4)	2 (10)	**>0.99**	10 (11.1)	7 (13.7)	**0.78**
Diabetes	14 (9.9)	11 (9.1)	3 (15)	**0.42**	6 (6.7)	8 (15.7)	**0.14**
Chronic Renal Failure	3 (2.1)	2 (1.6)	1 (5)	**0.37**	2 (2.2)	1 (2)	**>0.99**
Liver Disease	6 (4.3)	6 (4.9)	0	**0.59**	4 (4.4)	2 (3.9)	**>0.99**
Other	31 (22.0)	27 (22)	4 (20)	**>0.99**	20 (22.2)	11 (21.6)	**>0.99**
**Primary tumor**							
Colon-Rectum	57 (40.4)	51 (42.5)	6 (30)	**0.33**	36 (40)	21 (41.2)	**>0.99**
Melanoma	12 (8.5)	12 (9.9)	0	**0.21**	6 (6.7)	6 (11.8)	**0.35**
Uterus	4 (2.8)	3 (2.5)	1 (5)	**0.46**	4 (4.4)	0	**0.30**
Breast	6 (4.3)	4 (3.3)	2 (10)	**0.20**	5 (5.6)	1 (2)	**0.41**
Head-Neck	6 (4.3)	6 (4.9)	0	**0.59**	4 (4.4)	2 (3.9)	**>0.99**
NSCLC	12 (8.5)	9 (7.4)	3 (15)	**0.37**	5 (5.6)	7 (13.7)	**0.12**
Kidney	16 (11.3)	14 (11.6)	2 (10)	**>0.99**	11 (12.2)	5 (9.8)	**0.78**
Sarcoma	14 (9.9)	10 (8.3)	4 (20)	**0.11**	10 (11.1)	4 (7.8)	**0.77**
Other	14 (9.9)	12 (9.9)	2 (10)	**>0.99**	9 (10)	5 (9.8)	**>0.99**
**RT/CHT**							
Neoadjuvant	21 (14.9)	20 (16.5)	1 (5)	**0.30**	13 (14.4)	8 (15.7)	**>0.99**
Adjuvant	88 (62.4)	77 (63.6)	11 (55)	**0.47**	62 (68.9)	26 (51)	**0.05**
**Metastases**							
Synchronous	14 (9.9)	14 (11.6)	0	**0.22**	11 (12.2)	3 (5.9)	**0.38**
Size (mm)	14 (IQR 10–25)	13 (IQR 10–25)	16 (IQR 10–30)	**0.24**	13 (IQR 10–25)	16 (IQR 10–30)	**0.24**
**Number of Lesions**							
1	105 (74.5)	69 (76.7)	36 (70.6)	**0.65**	69 (76.7)	36 (70.6)	**0.65**
2	24 (17)	12 (13.3)	12 (23.5)		12 (13.3)	12 (23.5)	
≥3	12 (8.5)	9 (10)	3 (5.9)		9 (10)	3 (5.9)	
**Bilateral Nodules**	16 (11.3)	11 (9.1)	5 (25)	**0.053**	11 (12.2)	5 (9.8)	**0.79**
**DFI (Months)**	30 (IQR 17–56)	29 (IQR 16–49)	46 (IQR 22–121)	**0.07**	32.5 (IQR 18–61)	23 (IQR 14–49)	**0.24**

Values are expressed as n (%) or median (interquartile range). Abbreviations: ASA: American Society of Anesthesiologist Score, CHT: chemotherapy, DFI: disease-free interval, RT: radiotherapy.

**Table 2:** Operative approach and kind of resection.

Population	All 141 (100)	NLR < 4 121 (85.8)	NLR ≥ 4 20 (14.2)	*p*	CRP < 5 90 (63.8)	CRP ≥ 5 51 (36.2)	*p*
**Resection**							
Tumorectomy	3 (2.1)	3 (2.5)	0	**>0.99**	1 (1.1)	2 (3.9)	**0.29**
Wedge Resection	88 (62.4)	76 (62.8)	12 (60)	**0.46**	65 (72.2)	28 (54.9)	**0.04**
Segmentectomy	10 (7.1)	9 (7.4)	1 (5)	**>0.99**	6 (6.7)	4 (7.8)	**>0.99**
Lobectomy/bilobectomy	40 (28.4)	33 (27.3)	7 (35)	**0.79**	20 (22.2)	20 (39.2)	**0.03**
**Approach**							
VATS	78 (55.3)	72 (59.5)	6 (30)	**0.016**	57 (63.3)	21 (41.2)	**0.01**
**Time of Surgery (min)**	125 (IQR 85–190)	120 (IQR 80–190)	142 (IQR 117.5–182.5)	**0.20**	120 (IQR 80–170)	140 (IQR 95–205)	**0.21**
**Post-Resection Status**							
R0	135 (95.7)	115 (95.1)	20 (100)	**0.59**	86 (95.6)	49 (96.1)	**>0.99**

Values are expressed as n (%) or median (interquartile range). Abbreviations: VATS: video-assisted thoracic surgery, R0: no microscopic residual disease.

**Table 3:** Postoperative outcomes.

Population	All 141 (100)	NLR < 4 121 (85.8)	NLR ≥ 4 20 (14.2)	*p*	CRP < 5 90 (63.8)	CRP ≥ 5 51 (36.2)	*p*
**Length of Stay (days)**	5 (IQR 3–7)	5 (IQR 3–7)	5.5 (IQR 4–8)	**0.19**	5 (IQR 3–8)	5 (IQR 4–7)	**0.57**
**Complications**							
Total	17 (12.6)	13 (10.7)	4 (20)	**0.26**	9 (10)	8 (15.7)	**0.42**
Hemorrhage	2 (1.4)	2 (1.6)	0	**>0.99**	2 (2.2)	0	**0.53**
Persistent Air-Leak	6 (4.3)	5 (4.1)	1 (5)	**>0.99**	4 (4.4)	2 (3.9)	**>0.99**
Arrhythmia	3 (2.1)	1 (0.8)	2 (10)	**0.053**	2 (2.2)	1 (2)	**>0.99**
ARDS	1 (0.7)	0	1 (5)	**0.14**	0	1 (2)	**0.36**
Pneumonia	4 (2.8)	3 (2.5)	1 (5)	**0.46**	2 (2.2)	2 (3.9)	**0.62**
Other	7 (4.9)	6 (5)	1 (5)	**>0.99**	3 (3.3)	4 (7.8)	**0.25**

Values are expressed as n (%) or median (interquartile range). Abbreviations: ARDS: acute respiratory distress syndrome.

**Table 4:** Results of multivariate analysis.

Survival			Recurrence		
	HR	CI		SHR	CI
**Preop Neutrophils**	1.113	1.030–1.227	**Preop CRP**	1.015	1.008–1.022
**NLR**	1.893	0.846–4.327	**Age**	0.985	0.972–1.843
**Lung Comorbidity**	2.127	1.117–4.051	**Lung Comorbidity**	2.448	1.425–4.206
**Post-Op Pneumonia**	2.869	0.953–8.639	**N of Metastasis**	1.303	1.092–1.554
**Other Complications**	2.232	0.897–5.552	**Year of Surgery**	0.901	0.70–3.753
**Year of Surgery**	0.847	0.632–4.129			

CI: confidence interval; HR: hazard ratio; SHR: sub-hazard ratio.
